# Navigating the Challenges of Acanthamoeba Keratitis: Current Trends and Future Directions

**DOI:** 10.3390/life15060933

**Published:** 2025-06-10

**Authors:** Lanxing Fu, Joanna Wasielica-Poslednik, Gerd Geerling, Scott Robbie, Fabiana D’Esposito, Mutali Musa, Daniele Tognetto, Rosa Giglio, Caterina Gagliano, Marco Zeppieri

**Affiliations:** 1 Department of Ophthalmology, East Kent Hospitals University NHS Foundation Trust, Canterbury CT1 3NG, UK; l.fu@nhs.net; 2 Department of Ophthalmology, University Medical Center, Johannes Gutenberg University Mainz, 55131 Mainz, Germany; 3 Department of Ophthalmology, University Hospital Düsseldorf, Heinrich Heine University Düsseldorf, 40225 Düsseldorf, Germany; 4 Department of Ophthalmology, Guy’s and St Thomas’ NHS Foundation Trust, London SE1 7EH, UK; 5 Imperial College Ophthalmic Research Group (ICORG) Unit, Imperial College, London NW1 5QH, UK; 6 Department of Neurosciences, Reproductive Sciences and Dentistry, University of Naples Federico II, Via Pansini 5, 80131 Napoli, Italy; 7 Department of Optometry, University of Benin, Benin 300283, Nigeria; 8 Department of Medicine, Surgery and Health Sciences, University of Trieste, 34129 Trieste, Italy; 9 Department of Medicine and Surgery, University of Enna “Kore”, 94100 Enna, Italy; 10 Mediterranean Foundation “G.B. Morgagni”, 95100 Catania, Italy; 11 Department of Ophthalmology, University Hospital of Udine, 33100 Udine, Italy

**Keywords:** acanthamoeba keratitis, contact lens, keratitis, cornea, in vivo confocal microscopy

## Abstract

Acanthamoeba keratitis (AK) is a vision-threatening eye infection induced by the free-living species of the amoeba genus *Acanthamoeba*, presenting considerable therapeutic difficulties due to its frequently delayed diagnosis, chronic nature, and resistance to standard treatments. This review examines the changing landscape of AK, emphasizing recent developments in pathogenesis, diagnosis, and treatment. We examine the molecular pathways that enable *Acanthamoeba* invasion and persistence in the cornea, and how comprehending these processes can inform the creation of more effective treatment therapies. The review emphasizes current advancements in diagnostic methodologies, such as PCR-based tests and in vivo confocal imaging, which have enhanced early detection rates and diagnostic precision. Furthermore, we analyze contemporary treatment modalities, including antimicrobial therapy and surgical procedures, while recognizing the difficulties presented by antimicrobial resistance and the lack of standardized treatment protocols. This review seeks to deliver a thorough examination of AK, presenting insights into cutting-edge treatments and pinpointing essential areas for future research to address the persistent hurdles in controlling this potentially devastating ocular infection.

## 1. Introduction

*Acanthamoeba* spp. are unicellular protists identified in the 1930s and recognized as the causal agents of keratitis and amoebic granulomatous encephalitis [1,2]. They are free-living organisms found in the environment that mostly derive their sustenance from bacteria, hence enhancing soil mineralization [3]. *Acanthamoeba* have been extracted from freshwater, ocean water, air conditioning systems, humidifiers, dialysis machines, and surgical equipment, with *Acanthamoeba castellanii* being the main species. They are primarily linked to contact lens-related keratitis, although trauma, ocular surface illness, and environmental contamination may also be contributory factors to infection. Research has established a correlation between polluted household water sources, such as tap water, and acanthamoeba keratitis (AK) [4,5]. Radford et al. reported a ninefold elevated risk of developing AK in contact lens users in southern England compared with those in the north [6]. This is probably attributable to the greater incidence of residential hard water in comparison to soft water found in southern England. It was also shown that 88% (*n* = 93) of individuals diagnosed with AK were contact lens users [6]. A new systematic review and meta-analysis by Aiello et al. synthesized 105 pertinent studies published from 1987 to 2022 to present an updated global incidence of AK. The mean yearly center incidence exhibited considerable variation, with a median of 1.9 new AK cases per treatment center annually [7]. This variation included treatment centers, predominantly comprising tertiary eye care facilities. Consequently, a complicated interaction exists between local geography, environmental conditions, patient lifestyle factors, and variations in diagnostic methodologies that influence the occurrence of AK. This review aims to update the current understanding of the pathophysiology, clinical characteristics, diagnostic methods, and medical and surgical therapy of acanthamoeba keratitis.

## 2. Diagnosis of Acanthamoeba

### 2.1. Classification

Pussard and Pons (1977) classified the genus Acanthamoeba into three morphological groups according to cyst wall morphology [8]. This morphological classification, while historically significant, is constrained by the variety in cyst formations [8,9,10]. *Acanthamoeba castellanii* and polyphaga are the most common species associated with keratitis. Other *Acanthamoeba* species associated with keratitis are *Acanthamoeba culbertsoni*, *Acanthamoeba quina*, *Acanthamoeba hatchetti*, *Acanthamoeba rhysodes*, and *Acanthamoeba lugdunesis* [11]. Currently, classification is more commonly undertaken by a molecular phylogenetic-based approach [12]. This is because relying solely on morphological criteria can result in inconsistencies with species identification. Studies have shown disparities in cyst morphology amongst genetically identical isolates [13]. Based on a 5% minimum sequence divergence, over 23 genotypes have been identified through sequencing the 18S rRNA gene [10,14]. Isolates are classified on the gene sequence of the nuclear subunit 18S RNA [10,14]. Different species classified based on morphology can correspond with the same genotype using molecular techniques. The T4 genotype is the most predominant globally for keratitis, and there is no significant correlation between the described genotypes and clinical outcomes [15].

### 2.2. Pathogenesis

*Acanthamoeba* exist in two forms; the dormant cyst and the metabolically active trophozoite. The cyst form is double-walled, with a folded exocyst and a smooth endocyst [14]. Trophozoites multiply by mitosis and demonstrate active motility facilitated by lamellipodia. Acanthopodia are hypothesized to facilitate adhesion, although conclusive data are scarce. These acanthopodia help to bind the trophozoite to biological surfaces, and in a manner similar to the movement of macrophages, enable tissue invasion via paracellular penetration [11]. Adhesion molecules allow cell-to-cell contact, and promote interference with intracellular signaling pathways, which may lead to cellular apoptosis, phagocytosis, and secretion of toxins [16]. The ability to form cysts in an unfavorable environment allows *Acanthamoeba* to be viable almost indefinitely and contributes greatly to ongoing pathogenicity.

Pathogenesis of AK occurs when the trophozoite adheres to the corneal epithelium, facilitated by a mannose-binding protein that interacts with glycoproteins on the corneal epithelial cells. This is followed by the release of the protease MIP-133 that induces cell apoptosis and cytolysis, enabling deeper invasion into the corneal stroma. Therefore, MIP-133 and other proteases released by *Acanthamoeba* species can be potential targets for future therapeutics [16,17]. The use of contact lenses is an established risk factor for AK, and it has been shown to cause upregulation of mannosylated proteins on the corneal epithelium, thus making the corneal surface more susceptible to trophozoite binding [17]. The development of corneal abrasions also induces a change in mannosylated protein expression.

*Acanthamoeba*, like other amoebae, can internalize various microorganisms as endosymbionts and act as a reservoir for pathogens [18,19]. *Aspergillus* and *Fusarium* can utilize byproducts of amoebic metabolism as nutrition, as well as multiply without adverse effects on the host [20,21]. *Fusarium* and *Acanthamoeba* exist in similar environments, and the former happens to be the most common pathogen responsible for fungal keratitis, thereby contributing to enhanced pathogenicity during co-infection [20]. Iovieno et al. showed that out of 23 patients with confirmed AK, more than half of *Acanthamoeba* isolates contained one or more endosymbiont [18]. The formation of biofilms can further encourage excystation and resistance to disinfectants.

The human immune system responds vigorously to AK infection, which can result in worsening visual function. *Acanthamoeba* can evade the human complement system by the expression of complement-regulatory proteins, thereby deactivating the complement cascade, a key component in the body’s response to microbial infections [22]. The human adaptive immune system also responds to *Acanthamoeba*, with Alizadeh et al. showing that T-cells’ responses to *Acanthamoeba* antigens were present in 50% of asymptomatic patients. IgG serum antibodies specific to *Acanthamoeba* were expressed in 90% of patients with no known prior AK, due to the ubiquity of *Acanthamoeba* in the environment [23]. In animal models, secretory IgA antibodies have demonstrated protective effects during the initial stages of infection [23]. The ocular immune system does seem able to limit *Acanthamoeba* spread within a normal eye, as very few AK cases have been reported to involve the posterior segment [24,25,26].

### 2.3. Clinical Symptoms and Signs

**Figure 1:**
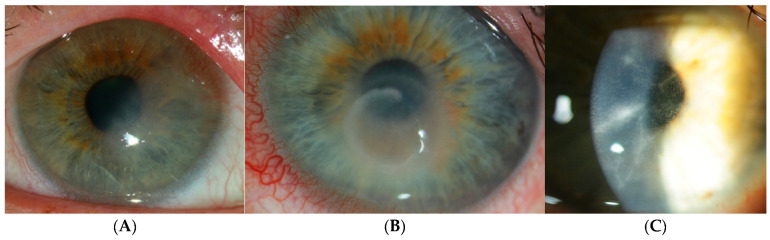
Traditional clinical manifestations of Acanthamoeba keratitis. (**A**) Epitheliopathy exhibiting irregular epithelial defects. (**B**) The immune ring is located in the mid-peripheral cornea. (**C**) Perineuritis is defined by radial infiltrates along corneal nerves.

Early AK is challenging to diagnose because the clinical signs are not pathognomonic and can mimic a host of other microbial keratitis. Early symptoms, such as “dirty epithelium” or pseudodendritic epitheliopathy (Figure 1), can easily be misdiagnosed as herpetic keratitis, which is the most common misdiagnosis in the early stage. Misdiagnoses can include herpetic keratitis, other types of microbial keratitis, or fungal infection [27]. Daas et al. showed that the correct AK diagnosis occurred on average after 2.8 ± 4.0 months following symptom onset [27].

Patients with early AK have nonspecific symptoms, such as tearing, redness, blurred vision, and mild discomfort. A distinguishing symptom with disease progression is intense pain, especially with increasing inflammation [28,29]. Most AK are unilateral, with only 7.5–11% reported bilateral AK, which mostly affects contact lens wearers [30,31,32,33]. Co-infection is also relatively common, such as with *Pseudomonas* species, which is the most common bacterial infection in contact lens wearers, and can affect up to 23% of AK cases [33,34]. Early clinical signs within the first fortnight of AK can include conjunctival follicular inflammation and subepithelial opacities, mimicking adenoviral conjunctivitis; epithelial defects and pseudodendritic epithelial changes that mimic herpes simplex keratitis [29,35]. Other signs can include microcysts and epithelial microerosions. Limbitis can occur due to direct *Acanthamoeba* invasion or as a result of the host s immune response.

Radial or perineural infiltrates may manifest adjacent to corneal nerves as the infection advances, resulting in severe pain (Figure 1C). Multiple stromal infiltrates can occur in AK, unlike the monofocal ones typically observed in bacterial keratitis. The characteristic Wessely immune ring can occur in other types of microbial and fungal keratitis, e.g., *Pseudomonas* [36,37,38,39]. AK disease progression has been categorized into five stages by Tu et al. based on clinical signs [37]. Complications can develop from *Acanthamoeba* infection and lead to secondary glaucoma from any associated raised intraocular pressure. This is due to cellular dysfunction of the trabecular meshwork, outflow blockage by inflammatory cells, fibrin, and posterior or peripheral anterior synechiae. Furthermore, biguanide therapy has been documented to induce a secondary increase in intraocular pressure, presumably attributable to its cytotoxic impact on the trabecular meshwork [12]. Anterior uveitis can occur in AK that is complicated by iris atrophy, anterior synechiae, and secondary cataract. In rare cases, inflammation can extend to the sclera and posterior segment [40,41,42]. Therefore, it is important to make a timely diagnosis and commence appropriate treatment in AK to preserve vision.

### 2.4. Current Techniques

Corneal scrapes for identification of isolates and cultures were, for a long time, considered the gold standard for detecting AK. When implemented, PCR significantly shortened the time needed to establish the correct diagnosis and reduced the need for corneal transplantation [13,43]. Corneal scrape sampling with bezel needles or cotton swabs is preferable to a blade for producing a positive sample [44,45]. The specimen sample is deposited onto a non-nutrient agar plate seeded with a layer of *Escherichia coli* or *Enterobacter cloacae* that serves as a nutrient source for the trophozoites. Monitoring of the agar plate for up to three weeks is necessary to confirm a negative result. Staining of the specimen can also help with diagnosis, usually with calcofluor white (CFW) or potassium hydroxide (KOH) wet mount; hematoxylin and eosin (H and E), periodic acid–Shiff (PAS), Gomori methanamine silver (GMS), and Gimenez stains are also used [46,47]. Of these stains, CFW has high specificity at 96% but a lower sensitivity of 71%, while KOH has a higher sensitivity of 91% and specificity of 100% [48,49]. In severe AK, the density of *Acanthamoeba* can be high, and can allow detection by direct microscopy. Trophozoites can measure between 15 and 45 µm with acanthopodia, and cysts are 12 to 25 µm with a double-layer wall [36]. The culture sensitivity can vary because the trophozoites and cysts can exist in deeper corneal layers and are not accessible by scraping. Hence, AK diagnosis usually consists of a combination of different diagnostic techniques (Table 1); the choice is dependent on clinician experience and availability [6,50].

**Figure 2:**
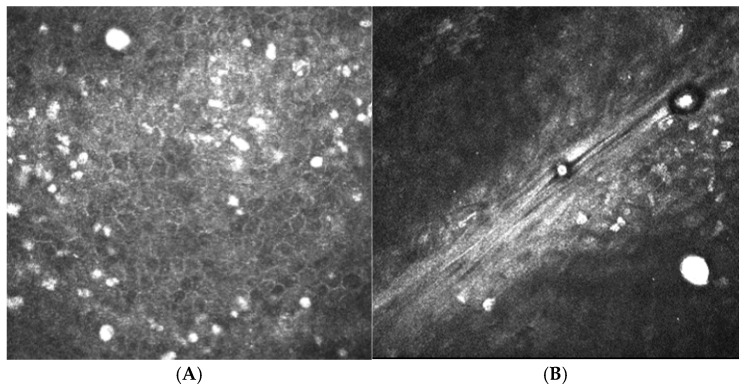
In vivo confocal microscopy images of Acanthamoeba cysts. (**A**) Cysts were identified in the superficial epithelium. (**B**) Cysts are seen in the anterior stroma. Cysts manifest as round, hyper-reflective entities ranging from 12 to 25 µm in diameter.

In vivo confocal microscopy (IVCM) can be used to image all layers of the cornea and is beneficial as a non-invasive method to detect *Acanthamoeba* (Figure 2). It is a rapid diagnostic method with 100% specificity and high sensitivity (85 to 100%) [51,52,53]. Most commonly used is the laser scanning model with a mountable objective system (Heidelberg Retinal Tomograph II with Rostock Corneal Module (HRT-RCM); Heidelberg Engineering GmbH, Heidelberg, Germany). Other IVCM models include the slit scanning confocal microscope (SSCM) Confoscan series (Nidek Technologies Padova, Padua, Italy) and the tandem scanning confocal microscope (TSCM). *Acanthamoeba* cysts may be identified as round, highly reflective structures measuring between 12 and 25 µm, and can appear in a “starry sky” pattern [54,55,56]. Trophozoites are more challenging to visualize on IVCM, as their appearance can be similar to keratocyte nuclei or leukocytes [52,57,58]. Other features include hyper-reflectivity, a signet ring, and spindle-shaped surrounding materials [59,60,61]. After topical corticosteroids are administered, the *Acanthamoeba* cysts can form into clusters, forming biofilms and resulting in a poorer outcome [62,63]. As the corneal tissue layers are damaged by AK, IVCM can detect changes in the morphology of keratocytes with reduced transparency of cellular structures [62,63,64]. The main limitations of the use of IVCM are the limited availability, the need for skilled operators, and the time to develop clinical expertise. It has limited ability to detect trophozoites, and current machines can only scan a small section of the cornea at a time. Therefore, it cannot be used in isolation as a diagnostic tool.

Another non-invasive imaging technique that can aid in AK diagnosis is anterior segment optical coherence tomography (AS-OCT). This modality can facilitate detection of perineuritis that manifests as reflective oblique bands deep within the corneal stroma [65]. This is particularly useful if co-existing stromal oedema and infiltrates. AS-OCT may also permit determination of the depth at which the perineuritis occurs and can help distinguish it from similar findings in herpetic keratitis that are located in the sub-epithelial stroma [66]. Current AS-OCT machines cannot reliably detect *Acanthamoeba* cysts or trophozoites, thereby limiting their usefulness in this regard.

PCR is one of the most expeditious diagnostic tools for the detection of Acanthamoeba genetic material [67]. PCR analysis amplifies a section from the 18S rRNA gene with reported sensitivity ranging from 84% to 100%, and results can be available within a matter of hours [48,68,69,70,71]. The main limitation with PCR detection is that specificity can be reduced when it detects non-viable genetic material.

Impression cytology has been used successfully as a relatively non-invasive technique to detect other forms of microbial keratitis [72]. Superficial corneal epithelial cells are deposited onto the impression membrane with nitrocellulose filters, after which staining with CFW or PAS is undertaken to detect *Acanthamoeba* cysts. This diagnostic tool has high specificity for AK, but is not able to detect cysts located deeper in the cornea [73].

## 3. Management

### 3.1. Medical Treatment

The treatment for AK is often prolonged because the cystic form has significant drug resistance and can persist for months within the ocular tissues. A typical course of treatment includes a combination of different drugs (Table 2), with the most frequent being biguanides and diamidines [74]. The frequency of topical drug administration is hourly for the first few days and then gradually tapered over the next few weeks, dependent on the clinical response. The entire duration of treatment can range from 3 months to over 12 months.

Biguanides are effective against both AK forms, with polyhexamethylene biguanide (PHMB) and chlorhexidine binding to the ostiole mucopolysaccharide, enabling binding to the cell membrane’s phospholipid bilayer. This leads to increased membrane permeability and cell lysis [75]. Chlorhexidine is a smaller molecule compared to PHMB, which facilitates deeper penetration into the stromal tissue, and has been found to have comparative outcomes as a monotherapy compared with biguanides [75]. In vitro and in vivo topical effectiveness of the biguanides can differ [30]. PHMB concentrations from 0.02% to 0.06% and chlorhexidine 0.02% to 0.2% are currently most widely available [30]. The minimal cysticidal concentration (MCC) of PHMB and chlorhexidine is 100 times less than currently available topical preparations, but resistant cases require higher concentrations [76]. Studies have demonstrated that biguanide monotherapy for treatment of early AK is comparable to combination therapy with a biguanide and diamidine, which is appealing because of better patient compliance and reduced costs [75,77]. Recently, Dart et al. performed a phase 3 randomized controlled trial showing non-inferiority of PHMB 0.08% monotherapy treatment compared with PHMB 0.02% and propamidine in 127 AK patients [77]. A retrospective study of 227 patients concluded that PHMB 0.02% monotherapy had better outcomes compared with other treatment modalities [78]. Side effects of biguanide therapy include toxic keratopathy and secondary raised intraocular pressure [11].

Aromatic diamidines such as hexamidine and propamidine both have cystostatic activity but are not cysticidal, so they cannot be used as monotherapy [79]. Both diamidines are cationic surface-active molecules and increase cell permeability, causing protein and enzyme denaturation [76]. Like biguanides, diamidines can cause ocular surface toxicity such as corneal epitheliopathy. Combination therapy with biguanides has an efficacy of 78% with PHMB and 86% with chlorhexidine [75]. Biguanides have cysticidal activity and hence are considered a first-line therapy for AK.

Antifungal drugs such as voriconazole and posaconazole inhibit ergosterol synthesis, an essential element of the Acanthamoeba cell membrane [80]. Tu et al. showed complete resolution of two patients with recalcitrant AK with oral voriconazole [81]. Musayeva et al. found encouraging results with the use of triple therapy (voriconazole 1%, propamidine isethionate 0.1%, and PHMB 0.02%) in 28 patients, with 50% of them having established or advanced AK (ring infiltrates, glaucoma, endothelial deposits, anterior chamber inflammation, and scleritis) [82].

Miltefosine has shown mixed results in AK treatment. Bagga et al. conducted a pilot study with topical miltefosine 65 μg/mL that showed good in vitro susceptibility but poor clinical efficacy [83]. However, Thulasi et al. found that miltefosine successfully treated 15 AK patients with refractory disease, albeit with significant inflammation necessitating topical steroids in two-thirds of the cohort [84]. The variable response could be due to differences in therapy duration, drug formulation, and severity of AK. Certainly, more research into different therapy avenues is needed.

### 3.2. Use of Corticosteroids and NSAIDs

Dormant Acanthamoeba cysts can exist in the corneal stroma indefinitely and are a risk for recurrent AK [29]. Corticosteroids have been used both topically and systemically to manage the significant inflammatory response in AK. Dexamethasone treatment has been shown to activate trophozoites and a 4- to 6-fold increase in excystment, leading to enhanced cytolysis of host corneal cells [85]. This phenomenon has been shown in animal infection models. It is supported by clinical research that shows AK patients who inadvertently receive corticosteroids before anti-amoebic treatment have poorer outcomes compared with those who receive corticosteroids after anti-amoebic treatment [86].

The use of corticosteroids, therefore, remains a significant topic of debate, as it can mask clinical signs of trophozoite proliferation and cyst formation [87]. They are not generally used in early AK disease stages, but rather are initiated in eyes with persistent severe inflammation involving the cornea, limbus, and sclera, along with non-steroidal anti-inflammatory drugs (NSAIDs) to aid disease resolution. Concurrent administration of anti-amoebic agents is recommended. A recent phase 3 randomized controlled trial incorporated adjunctive topical steroid therapy in the treatment protocol, with withdrawal at baseline and initiation after 3 weeks of anti-amoebic therapy at the discretion of the clinicians [77]. The trial results indicated a cure rate of 86% with PHMB 0.08% monotherapy following the specified treatment protocol [77]. A multi-center randomized clinical trial has recently begun recruitment to assess the efficacy of topical corticosteroids in treating AK and its impact on vision after six months. The results are expected to influence clinical practice [88].

### 3.3. Surgical Options

AK infections that are resistant to treatment and/or pose a risk of corneal melt may prompt consideration of emergency keratoplasty, either deep anterior lamellar keratoplasty (DALK) or penetrating keratoplasty (PK). The role and timing of keratoplasty surgery in AK management are uncertain. Some studies have recommended early keratoplasty surgery within the first 30–60 days of AK symptoms alongside anti-amoebic treatment [89,90]. Sarnicola et al. performed DALK in 11 AK patients who were not responsive to medical treatment and had no episodes of AK recurrence or graft rejection postoperatively [90]. However, histologic examination found that deep surgical margins were not disease-free in two cases. DALK has reduced endothelial graft rejection risks compared with PK, but is technically more challenging to perform and will be less efficacious in removing the AK infection. Therapeutic PK à chaud in the acute setting is the surgical technique of choice to reduce the risk of scleral extension, as well as for severe abscess or perforation [91]. These eyes carry a poor prognosis, with AK recurrence post-keratoplasties occurring within the first two weeks postop [92]. The recurrence rate is dependent on the type of keratoplasty performed: 19% for DALK, 17% for PK, and 10% for elective optical keratoplasty, i.e., performed late after AK to remove a dense corneal scar [89]. Recurrence of AK is a result of cyst reactivation in the host recipient tissues and subsequent colonization of the grafted cornea [91]. A minimum 1 mm margin of visible healthy tissue is recommended along with several months of anti-amoebic treatment to reduce the risk of AK recurrence preceding and following surgery [7,30]. Amniotic membrane transplantation can be an adjuvant treatment along with therapeutic keratoplasty to encourage epithelial and stromal tissue healing and is anti-inflammatory. Multiple amniotic membrane grafts may be required for complete recovery, and optical keratoplasty may be necessary for visual restoration [7,55,91,93].

**Figure 3:**
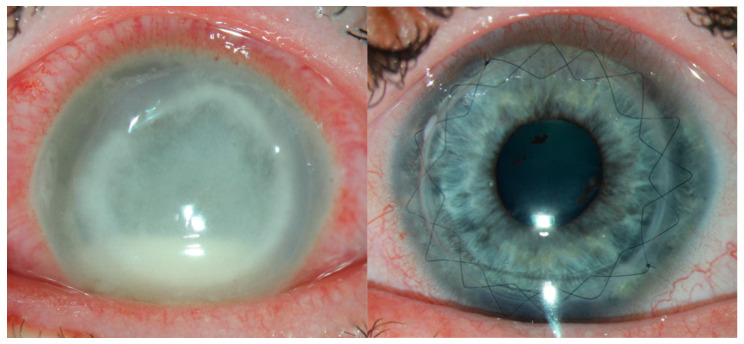
Keratoplasty is sometimes needed both in the short term, if there is perforation, and long term for visual rehabilitation. This patient had severe, advanced acanthamoeba (**left**) and recovered vision well following optical penetrating keratoplasty after 6 months of medical treatment (**right**).

Once the AK has resolved, elective keratoplasty is useful for treating corneal scarring and restoring vision (Figure 3). Szentmáry et al. demonstrated that 94% of AK patients who received an optical keratoplasty had a clear graft during follow-up, and 40% had a visual acuity that exceeded 20/30 [91]. Optical keratoplasty has been shown to have the lowest rate of AK recurrence, so medical management should be prioritized until AK has been eradicated from the eye.

## 4. Future Directions

### 4.1. Use of Artificial Intelligence in AK Diagnosis

The diagnosis of AK is based on clinical findings (with a high index of suspicion), corneal tissue culture and biopsy, confocal microscopy, and PCR. These diagnostic techniques rely on experience, time, and cost and result in tissue loss. In low-resource or remote settings, diagnosis can be particularly challenging. Artificial intelligence (AI) models have been utilized in the diagnostic workup of AK, especially in the realms of image analysis, early detection with IVCM image analysis, and within decision support systems to generate a risk score. This enables less dependence on human expert interpretation, allowing better accessibility.

AI models can analyze IVCM images to detect patterns indicative of AK, potentially leading to earlier and more accurate diagnoses. Essalat et al. utilized a deep learning model that showed good ability to distinguish between *Acanthamoeba* (sensitivity of 91% and specificity of 98%) and fungal keratitis (sensitivity of 97% and specificity of 96%) [94]. A recent retrospective cohort study comprising 3312 IVCM images from 17 culture-positive AK patients was used to train a deep learning model that resulted in sensitivity and specificity of 76%, respectively [95]. Another study by Koyama et al. applied techniques of facial recognition on slit-lamp images (different angles, resolution, and levels of illumination) to diagnose *Acanthamoeba* with an accuracy rate of 98% [96]. Combining several different AI models can improve diagnostic performance. Zhang et al. utilized combined AI models and had an accuracy of 83.8% for AK diagnosis, which was better than the accuracy of their study’s invited corneal experts [97]. Given the diversity of AK presentation globally, more validation is required across diverse datasets to improve AI models for diagnosis and treatment. Future research will be aimed toward developing AI tools that can be utilized in the process of early diagnosis and treatment, and determining how to integrate these into the clinical workflows to facilitate daily practice.

### 4.2. Standardized Treatment Protocols

Protocolized treatment is established in medicine for severe infections such as sepsis, with clear improvement in clinical outcomes for patients [98]. Currently, it is rare outside of randomized controlled trials for protocolized treatments to be given to keratitis patients. The prolonged treatment course for AK, with its individualized treatment that is practitioner-dependent and with no standardized termination protocols, makes outcome evaluation challenging. Sharma et al. published a protocol for fungal keratitis (Topical, Systemic, and Targeted Therapy; TST) that has demonstrated good outcomes compared to individualized treatment [99]. Recently, Dart et al. showed a 1.59-fold improvement in clinical outcomes for AK patients using a protocol-based treatment plan compared with individualized treatment [100]. With the advent of standardized preparations of anti-amoebic drugs becoming available, using standardized treatment protocols will allow better evaluation of treatment outcomes.

### 4.3. Cross-Linking

Corneal collagen cross-linking has been applied to treat microbial keratitis, including AK (PACK-CXL; photoactivated chromophore for keratitis corneal cross-linking). The proposed mechanism of action entails the impairment of the Acanthamoeba cell membrane and nucleic acids by reactive oxygen species [101]. Atalay et al. established that PACK-CXL with riboflavin 0.1% and 0.25% exhibited no amoebicidal impact, whereas rose-bengal-mediated PACK-CXL did have in vitro anti-amoebic action against *Acanthamoeba castellanii* [102]. In a subsequent animal model, the same authors showed that rose bengal PACK-CXL effectively decreased the AK clinical severity and AK load [103]. A recent meta-analysis comprising 46 studies totaling 435 patients concluded that PACK-CXL helped to expedite healing as an adjuvant treatment. Still, there was insufficient evidence for its primary use in AK [104]. Histologically, it has been shown that, despite PACK-CXL treatment, AK cysts can persist within the corneal stroma [105]. Hence, PACK-CXL is not routinely used by AK.

## 5. Conclusions

AK remains a significant clinical challenge due to its diagnostic complexity, treatment resistance, and potential for vision-threatening complications. Its association with contact lens use and environmental exposure underscores the importance of public awareness and preventive strategies. Accurate and early diagnosis, utilizing a combination of traditional methods and emerging technologies such as in vivo confocal microscopy and PCR, is critical for optimal outcomes. Advances in artificial intelligence offer promising support in image analysis and diagnostic decision-making, especially in resource-limited settings. While medical management with biguanides remains the mainstay, adjunctive therapies like PACK-CXL and surgical interventions are valuable in select cases. The development of standardized treatment protocols could streamline care and improve patient outcomes. Continued research into therapeutic agents, diagnostic tools, and AI integration will be essential to addressing the burden of AK and enhancing vision preservation globally. A multidisciplinary and evidence-based approach remains vital in managing this complex ocular infection.

## Figures and Tables

**Table 1:** Advantages and disadvantages of the current diagnostic techniques for Acanthamoeba keratitis.

Diagnostic Technique	Advantages	Disadvantages	Target Detected
**Corneal scrape/** **Culture**	Good sensitivity High specificity The only method for detecting viable *Acanthamoeba*	In co-infection, there is a risk of misdiagnosis. Corneal scrapes cannot access deeper stroma, where AK can infiltrate.	Cysts and trophozoites
**PCR**	High sensitivity Rapid turnaround	False positives due to amplification of non-viable Acanthamoeba genomes.	Cysts and trophozoites
**In Vivo Confocal** **Microscopy (IVCM)**	Sensitivity and specificity are both high; a rapid and non-invasive test	Significant learning curve required and operator-dependent. A small area of the cornea is covered per scan, and there is limited availability.	Primarily cysts
**Impression Cytology**	High specificity	Requires expertise in cytopathology and specialized stains. Cannot detect deep Acanthamoeba cysts.	Superficial cysts only
**AS-OCT**	Useful for differential diagnosis, non-invasive	Current machines cannot detect Acanthamoeba trophozoites and cysts directly.	Indirect structural signs only

PCR = polymerase chain reaction; IVCM = in vivo confocal microscopy; AS-OCT = anterior segment optical coherence tomography.

**Table 2:** Summary of anti-amoebic agents used in the medical treatment of Acanthamoeba keratitis.

Treatment Category	Agent(s)	Main Point	Cidal/Static Activity and Target
**Biguanides**	Polyhexamethylene biguanide (PHMB)	First-line therapy; effective as monotherapy or in combination	**Cidal**—cysts and trophozoites
	Chlorhexidine	Smaller molecule allows deeper corneal penetration; comparable outcomes to PHMB.	**Cidal**—cysts and trophozoites
**Aromatic Diamidines**	Propamidine, Hexamidine	Used in combination with biguanides; not effective as monotherapy	**Static**—primarily trophozoites
**Antifungal Agents**	Voriconazole, Posaconazole	Antifungal agents with anti-amoebic activity; used in refractory or co-infected cases	**Cidal**—primarily cysts
**Phosphocholine Analog**	Miltefosine	Used in refractory cases; topical and oral forms trialed; limited accessibility	**Cidal**—cysts and trophozoites (variable efficacy)

## Data Availability

No new data were created or analyzed in this study. Data sharing is not applicable to this article.
